# The Amino Acid-Mediated TOR Pathway Regulates Reproductive Potential and Population Growth in *Cyrtorhinus lividipennis* Reuter (Hemiptera: Miridae)

**DOI:** 10.3389/fphys.2020.617237

**Published:** 2020-11-30

**Authors:** Haowen Zhu, Sui Zheng, Jinming Xu, Qing Wu, Qisheng Song, Linquan Ge

**Affiliations:** ^1^School of Horticulture and Plant Protection, Yangzhou University, Yangzhou, China; ^2^Division of Plant Sciences, University of Missouri, Columbia, MO, United States

**Keywords:** *Cyrtorhinus lividipennis*, target of rapamycin, amino acid, fecundity, population growth

## Abstract

The predatory mirid bug, *Cyrtorhinus lividipennis* Reuter, feeds on brown planthopper (BPH) eggs that are deposited on rice and gramineous plants surrounding rice fields. The development and reproduction of *C. lividipennis* are inhibited by feeding on BPH eggs from gramineous species, and the underlining regulatory mechanism for this phenomenon is unclear. In the present study, HPLC-MS/MS analysis revealed that the concentrations of six amino acids (AAs:Ala, Arg, Ser, Lys, Thr, and Pro) were significantly higher in rice than in five gramineous species. When *C. lividipennis* fed on gramineous plants with BPH eggs, expression of several genes in the target of rapamycin (TOR) pathway (*Rheb*, *TOR*, and *S6K*) were significantly lower than that in the insects fed on rice plants with BPH eggs. Treatment of *C. lividipennis* females with rapamycin, dsRheb, dsTOR, or dsS6K caused a decrease in *Rheb*, *TOR*, and *S6K* expression, and these effects were partially rescued by the juvenile hormone (JH) analog, methoprene. Dietary dsTOR treatment significantly influenced a number of physiological parameters and resulted in impaired predatory capacity, fecundity, and population growth. This study indicates that these six AAs play an important role in the mediated-TOR pathway, which in turn regulates vitellogenin (Vg) synthesis, reproduction, and population growth in *C. lividipennis*.

## Introduction

The mirid bug *Cyrtorhinus lividipennis* (Reuter; Hemiptera: Miridae) is a natural predator of rice planthoppers. Predation by *C. lividipennis* modulates the population densities of several planthoppers in rice fields, including the brown planthopper (BPH), *Nilapavata lugens* (Katti et al., 2007; Sigsgaard, 2007). *Cyrtorhinus lividipennis* nymphs and adults mainly consume planthopper eggs, nymphs, and adults for growth and development (Reyes and Gabriel, 1975). *Cyrtorhinus lividipennis* also preys on lepidopteran pests including *Chilo suppressalis*, *Cnaphalocrocis medinalis*, and *Sesamia inferens* (Zhu and Chen, 1981).

The feeding and reproduction of insect species are closely correlated with nutritional factors (Yu et al., 1996). *Cyrtorhinus lividipennis* is an unusual species that exhibits both herbivorous and predatory feeding habits (Bentur and Kalode, 1985). When *C. lividipennis* consumed a diet of planthoppers that inhabited gramineous species such as *Echinochloa glabrescens*, *Leptochloa chinesis*, *Digitaria ciliaris*, *Cyodon dactylon*, and *Eleusine indica* growing on the bunds surrounding rice fields, their development and reproduction were reduced in comparison to those consuming planthoppers (such as BPH eggs) on rice plants (Pomari-Fernandes et al., 2015), indicating that nutrition from rice plant is beneficial to the predator. When *C. lividipennis* was fed on eggs of the rice meal moth, *Corcyra cephalonica* (Lepidoptera: Pyralidae), the predator successfully reached the adult stage (Murugan and George, 1992). Similarly, *Telenomus remus* (Hymenoptera:Platygastridae) showed normal development when fed on *C. cephalonica* eggs, suggesting that *C. cephalonica* is a promising factitious host (Matteson, 2000). Collectively, these results indicate that *C. cephalonica* eggs or planthopper eggs/nymphs from rice plants provide adequate nutrients for successfully rearing predatory insects.

Predation capacity is influenced by changes in the environment, host morphology, and nutrient composition (Wakde, 1995; Laba and Heong, 1996; Cortesero et al., 2000). With respect to the latter category, proteins, free amino acids (AAs), sugars, lipids, inorganic salts, and vitamins provide energy and compounds needed for insect growth and development (Shah et al., 2005). AAs are among the most important nutrients and participate in various metabolic processes in insects (Sogawa, 1972); furthermore, AAs were shown to significantly impact the balance between fecundity and lifespan in *Drosophila* (Grandison et al., 2009). Our recent transcriptomic analysis revealed that, when newly-emerged *C. lividipennis* fed on BPH eggs from any of the five gramineous species mentioned above, the expression levels of the *TOR* and *S6K* genes in the target of rapamycin (TOR) pathway were significantly downregulated, compared to those feeding on BPH eggs from rice plants or *C. cephalonica* eggs (data not shown).

The TOR signaling pathway transduces nutritional signals that stimulate vitellogenin (Vg) synthesis in insect fat body, which subsequently activates egg development (Kim and Guan, 2011; Roy and Rikhel, 2011; Perex-Hedo et al., 2013). In *Aedes aegypti*, the TOR signaling pathway genes (e.g., *S6K*, *Rheb*, and *TSC2*) relay nutritional information during egg development (Hansen et al., 2004, 2005; Roy and Rikhel, 2011). Furthermore, the knockdown of *TOR* gene in *A. aegypti* reduced *Vg* transcription and resulted in reduced numbers of deposited eggs, suggesting that the TOR pathway is a critical component in Vg biosynthesis and the maturation of oocytes (Hansen et al., 2004, 2005; Roy and Rikhel, 2011). In *Blattella germanica*, the TOR pathway reportedly links nutritional signals with juvenile hormone (JH) and Vg synthesis (Maestro et al., 2009). An analogous relationship between the TOR signaling pathway and JH synthesis was identified in BPH (Lu et al., 2016). Collectively, these studies indicate that Vg transcription and egg maturation are controlled by endocrine hormones and the TOR signaling pathway in insects.

In the present study, we explore the question of why a diet of BPH eggs from gramineous plants is an unsuitable food source for *C. lividipennis*. This study illuminates one of the underlying regulatory mechanisms of this phenomenon and provides valuable information regarding the role of six AAs in the reproduction of *C. lividipennis.*

## Materials and Methods

### Plant Materials and Insects

The BPH-susceptible rice cultivar Ningjing 4 was used in this study. Seeds were planted in a cement pool, and seedlings were transferred into plastic pots and arranged in hills at the six-leaf stage as previously described (Lu et al., 2017; Ge et al., 2020a). All experiments utilized rice at the tillering stage (40 days).

The seeds of gramineous plants, including *L. chinesis*, *D. ciliaris*, *C. dactylon*, and *E. indica*, were purchased from Jiangsu Leerda Seed Industry Co., Ltd. (LiYang, Jiangsu). Seeds of the barnyard grass *E. glabrescens* were collected from rice fields located at the Yangzhou University Farm. Seeds were sown in plastic pots (16 cm diameter × 15 cm high). The six gramineous plants were used in experiments 40 days after germination because plenty of BPH eggs were laid on these plants at this time. Each newly-emerged *C. lividipennis* female was offered 10 rice plants with BPH eggs or 50 *C. cephalonica* eggs as food sources.

The BPH strain utilized herein was provided by the China National Rice Research Institute (Hangzhou) and reared as described previously (Ge et al., 2020a). *Cyrtorhinus lividipennis* was obtained from rice plants cultivated in Yangzhou and maintained on Ningjing 4 rice containing *N. lugens* eggs or nymphs.

### Reagents and Quantitation of Free Amino Acids

Amino acids, vitamins, inorganic salts, and other reagents were purchased from Sinopharma Chemical Reagent Co. Ltd. (Shanghai, China). Stems (0.1 g fresh weight) were removed from rice (tillering stage) and five gramineous species at 40 days after germination, respectively, and transferred to 1 ml of 0.01 N HCl. Suspensions were allowed to settle for 15 min at ambient temperature, and a 0.5 ml volume was centrifuged at 10,000 × g. AAs were extracted using the EZ:Fast Free AA Kit (Phenomenex, CA, United States). HPLC-MS/MS analysis of free AAs in extracted samples was conducted as described (Florencio-Ortiz et al., 2018). AA standards were supplied with the EZ:Fast kit, and calibration curves were calculated for each AA. Data were analyzed with Agilent 5975 software.

### Artificial Diets for *C. lividipennis*

Artificial diets were formulated as described previously (Ge et al., 2020b). Compounds were dissolved in double-distilled water (ddH_2_O), and the pH was adjusted to 6.4 with 4% KOH in a total volume of 100 ml. Diets were prepared with AAs (+6 AAs:Ala, Arg, Lys, Ser, Pro, and Thr), without AAs (−6 AAs:−Ala, −Arg, −Lys, −Ser, −Pro, and −Thr), or lacking individual AAs (−1 AA:−Ala, −Arg, −Lys, −Ser, −Pro, or −Thr). Glass cylinders (7.5 × 1.2 cm) were used as feeding chambers. A 50 μl aliquot of the artificial diet was sandwiched between layers of a Parafilm membrane as described previously (Ge et al., 2020a). The diet capsule was replaced on alternate days, and 200 μl ddH_2_O was added daily to maintain RH (He et al., 2014). A black cotton cloth was used to exclude light from the cylinders; however, the open end containing the diet capsule was left uncovered to provide light. *Cyrtorhinus lividipennis* feeding (*n* = 10 first instar individuals) was facilitated by puncturing the inner Parafilm membrane, and 10 chambers were used to rear nymphs in each treatment. Rearing was conducted in growth chambers maintained at 26 ± 2°C, 80% RH with a 16L:8D photoperiod. Mortality was recorded on alternate days.

### dsRNA Preparation and Microinjection

*Rheb*, *TOR*, and *S6K* were amplified from cDNA using primer sets Rheb-F/Rheb-R, TOR-F/TOR-R, and S6K-F/S6K-R (Supplementary Table S1). The green fluorescent protein (*GFP*) gene encoding GFP was used as a negative control; this was amplified as a 688-bp fragment from pHT3AG with primers GFP-F and GFP-R. The reaction conditions were as follows: 35 cycles at 95°C for 30 s, 60°C for 30 s, and 72°C for 45 s, with a final extension at 72°C for 10 min. Cloned PCR products were used as templates to re-amplify the genes for dsRNA synthesis using the following primer sets: Rheb-T_7_F/Rheb-T_7_R, TOR-T_7_F/TOR- T_7_R, S6K-T_7_F/S6K-R, and GFP-T_7_F/GFP-T_7_R (Supplementary Table S1). A DNA gel purification kit (Omega Bio-tek, Doraville, GA, United States) was used to purify PCR products. The T_7_ RiboMAX™ Express RNAi System (Promega, Madison, WI, United States) was used to generate dsRNAs by *in vitro* transcription. The dsRNA products were dissolved in diethyl pyrocarbonate (DEPC)-treated water at 5 μg/μl and stored at −80°C.

Fifth instar *C. lividipennis* nymphs were anesthetized with CO_2_ prior to dsRNA injection (Lin et al., 2016). Injection of the intra-thoracic region was carried out using a Nanoject II microinjection device (Drummond Scientific, Broomall, PA, United States; Liu et al., 2010). dsRNA (50 ng in 50 μl) was injected into fifth instar nymphs; after a 2 h recovery period, nymphs were allowed to feed on the complete artificial diet. Insects were collected at 2 days after emergence (DAE) and used for total RNA extraction.

### Methoprene and Rapamycin Treatments

A stock solution (100 ng/nl) of the JH analog (JHA) methoprene (Sigma-Aldrich, St. Louis, MO, United States) was prepared in acetone as described (Ge et al., 2020a) and diluted to 10 μg/μl in acetone and ddH_2_O (1:10 v/v). Fifty nanoliters of 1.0 ng/nl JHA solution was topically applied to the dorsal side of newly emerged female as described (Ge et al., 2020a). Methoprene-treated *C. lividipennis* females were transferred into glass cylinders and maintained on normal artificial diets. *C. lividipennis* females were collected 48 h after topical application; quantitative real-time PCR (qPCR) was used to evaluate the expression of *Rheb*, *TOR*, *S6K*, and *Vg*, and immunoblotting was used to detect phosphorylation level of S6K and Vg. Treatments and controls consisted of three independent biological replicates.

Rapamycin (Sigma-Aldrich St. Louis, MO, United States) was dissolved in ethanol (Lu et al., 2016). The abdominal region of fifth instar nymphs was injected with a 100 nl solution of 2.0 nM rapamycin, whereas negative controls were injected with an equal volume of ethanol. The nymphs were supplied with the complete artificial diet containing AAs and reared until adults emerged. Newly emerged females were collected and treated with methoprene. The expression of *Rheb*, *TOR*, *S6K*, and *Vg* were determined using qPCR, and the phosphorylation of S6K and Vg were determined by western blot (WB) analysis 48 h after the topical application of JHA methoprene. Treatments and controls consisted of three independent biological replicates, with 15 females for each replicate.

### Quantitative Real-Time PCR

Trizol reagent (Invitrogen, Carlsbad, CA, United States) was used to extract RNA from *C. lividipennis*. cDNA was synthesized with PrimeScript RT Reagent Kit and gDNA Eraser (TakaRa Beijing, China). cDNA was synthesized in 20 μl reaction volumes at 37°C for 15 min in a mixture containing random hexamers and oligo dT primers.

Quantitative real-time PCR was conducted in a 7,500 real-time PCR system (Bio-Rad Co. Ltd., California, United States) in a 96-well format with SYBR Premix^EX^ Taq Kit (TakaRa, Tokyo, Japan). Reactions contained cDNA template (1 μl), SYBR master mix (5 μl), primers (0.4 μl/per primer at 10 μmol), and ddH_2_O (3.2 μl). The qPCR program was as follows: 95°C for 30 s, followed by 35 cycles of 95°C for 5 s, 60°C for 15 s, and 72°C for 30 s. Reactions were normalized using β-actin (EU179847), and the 2^−ΔΔct^ method was used to obtain relative mRNA expression levels (Livak and Schmittgen, 2001). The primers used for qPCR are shown in Supplementary Table S1.

### Protein Extraction and Determination

Soluble proteins were extracted from ovaries and fat bodies of 50 dsTOR-treated females and 50 dsGFP-treated control females at 2 DAE as described by Ge et al. (2010). The Bradford method (Bradford, 1976) was used to determine protein concentrations. *A*_595_ values were measured by UV spectrophotometry, and protein content was determined with a standard curve of BSA (Shanghai Biochemistry Research Institute, Shanghai, China). Treatments and controls consisted of three independent biological replicates.

### Female Body Mass and Isolation of Ovaries

The weights of 10 dsTOR- and 10 dsGFP-treated females (control) were recorded at 2 DAE. Treatments and controls consisted of three independent biological replicates (*n* = 10, 10 BPH females for each replicate, *N* = 3, 3 replicates).

Ovaries (*n* ≥ 10 mated females from dsTOR- and dsGFP-treatments at 7 DAE) were isolated in 10 mM phosphate buffered saline (PBS; pH 7.4), fixed in 3.8% formaldehyde, and washed with 0.2% Triton X-100 as described (Ge et al., 2019). After washing, images were photographed with a Leica DMR connected to a Fuji Fine PixS2 Pro digital camera (Germany).

### JH III and Ecdysteroid Titers

Titers of JH III and ecdysteroid were measured in adult females by HPLC/MS (Cornette et al., 2008; Giebbultowicz et al., 2008). 20-hydroxyecdysone and JH III standards were obtained from Sigma (St. Louis, MO, United States). Treatments and controls were replicated three times with five females for each replicate.

### Measurement of Predation Capacity in *C. lividipennis*

The predation capacity of *C. lividipennis* was measured as described by Base et al. (2002) with minor modifications. First instar nymphs of the prey, *N. lugens* (*n* = 20), were placed in glass tubes (3 × 25 cm) containing four 15 days-old rice seedlings of rice cv. “Ningjing 4,” which is susceptible to *N. lugens*. Newly emerged (<24 h) *C. lividipennis* adult females were individually placed into glass tubes, which were then sealed with nylon mesh. Surviving *N. lugens* nymphs were recorded daily, and the predation number per *C. lividipennis* was assessed based on survival counts of *N. lugens* nymphs at days 1 and 3. Treatments and controls consisted of five independent biological replicates.

### Western Blot Analysis

Immunoblotting was conducted as described previously with minor modifications (Ge et al., 2019). Fat bodies and ovaries were separately homogenized in lysis buffer (0.5 ml) supplemented with protease and phosphatase inhibitors (Lu et al., 2016) and incubated for 1 h at 4°C. Lysates were centrifuged and protein concentration in the resulting supernatant was analyzed using the Bradford method as described above. Total 30 μg protein was separated by 10% SDS-PAGE and transferred to PVDF membranes; the membranes were treated with a blocking solution [5% nonfat dry-milk in 10 mM Tris-buffered saline (TBS), pH 7.4, containing 0.5% TBS Tween-20 (TBST)] for 1 h and then incubated with primary antibodies at room temperature for 2 h. Anti-*phospho*-*p70 S6 kinase* (p70-Thr-389 S6K) polyclonal antiserum (1:5,000) was obtained from Cell Signaling Technology (Danvers, MA, United States), and anti-Vg antiserum (1:5,000) was prepared by Nanjing Kingsley Biotechnology Co. Ltd. (Nanjing, China). Antiserum to β-actin (1:5,000; Cell signaling Technology, Davers, MA, United States) served as a loading control. Membranes were washed with TTBS three times, 5 min each, and incubated within the TBS buffer containing goat anti-rabbit IgG secondary antibodies-conjugated to horseradish peroxidase (1: 8,000 dilution) for 1 h at room temperature. Reactive proteins were visualized with chemiluminescent substrates with the GBOX-Chemi XT4 system (Syngene, Cambridge, UK) as described previously (Lu et al., 2016).

### Immunofluorescence Microscopy

Ovarioles from *C. lividipennis* mated females were removed at 7 DAE, washed 3× in cold PBS (pH = 7.4, 10 mM), fixed in 4% paraformaldehyde for 2 h, and then washed with PBS three times. Ovaries were then washed three times in PBS containing 0.1% Triton X-100 (PBST), blocked in PBST containing 5% goat serum, and incubated with anti-Vg (1:500) as described previously (Ge et al., 2020a). After three washes with PBS, 5 min each, Alexa Fluor 488-labeled goat anti-rabbit secondary antibody (1:500; Beyotime, Shanghai, China) was added in PBST containing 2% goat serum and 3% BSA. After incubation at ambient temperature for 1 h at low light, nuclei were counterstained with 100 nM 4',6-diamidino-2-phenylindole (DAPI; Beyotime) for 10 min in PBST. Samples were placed on slides and washed in PBS three times with 5 min each. Fluorescence images were captured with a Zeiss LSM 780 confocal microscope (Carl Zeiss MicroImaging, Göttingen, Germany).

### Population Growth

Two groups were established to monitor population growth: dsGFP-treated females mated with untreated males (control group), and dsTOR-treated females mated with untreated males (treatment group). A randomized complete block containing five replicates was used as an experimental design. Newly emerged *C. lividipennis* (two pairs) were released on rice plants containing BPH eggs at the tillering stage (40 days after germination) and enclosed in nylon cylindrical cages as described (Ge et al., 2019). When the third instar nymphs of the next generation emerged (~25 days), groups were inspected daily and the third instar nymphs were counted; these nymphs were transferred to new plastic pots with tillering-stage rice plants. Nymphs were examined every 2 days until adults emerged; numbers of both sexes were recorded until the females died. Numbers of adults from the new generation and unhatched egg counts were used to calculate hatch rates and the ratio of adults/adults + unhatched eggs (Ge et al., 2019). The population growth index (PGI) was expressed by the ratio *N*1/*N*0; this was determined by dividing the total number (*N*1) of adults of next generation + unhatched eggs by the number of adults released (*N*0 = 8, 4 pairs).

### Statistical Analyses

Statistics were obtained with SPPS v. 18.0 (SPSS Inc., Chicago, IL, United States). The Shapiro-Wilk test was utilized to examine normality of data variances, and the *t*-test was performed to compare means of two variables. Fisher’s protected least significant difference (PLSD) test was applied to multiple comparisons of the means. Data points were considered significant at *p* < 0.05. Values were expressed as means ± SEM.

## Results

### Free Amino Acid Content in *C. lividipennis* Inhabiting Different Hosts

The concentrations of free AAs were measured for rice plant and five gramineous species (*C. dactylon*, *D. ciliaris*, *E. indica*, *E. glabrescens*, and *L. chinensis*; Table 1). The concentrations of six AAs (Ala, Arg, Lys, Pro, Ser, and Thr) were significantly higher in rice plant than that in gramineous species; exception was Lys, which was higher in *E. glabrescens* than rice (Table 1). The percent increases were 39–123% for Ala (*F* = 31786.7, *df* = 5, 17, *p* = 0.0001), 23–38% for Lys (*F* = 8350.9, *df* = 5,17, *p* = 0.0001), 67–147% for Pro (*F* = 2240.7, *df* = 5,17, *p* = 0.0001), 42–86% for Arg (*F* = 3688.8, *df* = 5,17, *p* = 0.0001), 40–82% for Ser (*F* = 696.9, *df* = 5, 17, *p* = 0.0001), and 41–86% for Thr (*F* = 424.4, *df* = 5,17, *p* = 0.0001).

**Table 1:** Free amino acid (AA) content (mg/100 g fresh weight) in rice and five different gramineous hosts as measured by HPLC-MS/MS.

Amino acid	*Oryza sativa*	*Digitaria ciliaris*	*Cynodon dactylon*	*Leptochloa chinensis*	*Eleusine indica*	*Echinochloa glabrescens*
Ala^*^	**14.70 ± 1.30**^a^	9.27 ± 0.97^c^	8.04 ± 0.85^c,d^	6.60 ± 0.6e	9.56 ± 0.93^c^	10.55 ± 1.32^b^
Cys	0.73 ± 0.09^b^	0.77 ± 0.08^b^	0.89 ± 0.13^a^	0.89 ± 0.09^a^	0.69 ± 0.08^c^	0.68 ± 0.07^d^
Asp	5.28 ± 0.43^a,b^	5.15 ± 0.49^a,b^	5.23 ± 0.59^a,b^	6.29 ± 0.87^a^	5.57 ± 0.37^a,b^	5.65 ± 0.18^a,b^
Glu	15.14 ± 1.25^a^	14.45 ± 1.02^a^	10.21 ± 1.12^c^	12.54 ± 1.04^b^	12.41 ± 1.17^b^	9.02 ± 1.25^d^
Phe	1.42 ± 0.12^b,c^	1.57 ± 0.09^b^	1.85 ± 0.11^a^	1.23 ± 0.20^c^	1.13 ± 0.11^c,d^	1.20 ± 0.01^c^
Gly	10.37 ± 1.22^a^	10.15 ± 1.06^a^	9.88 ± 1.31^a^	9.99 ± 1.13^a^	8.35 ± 1.02^a,b^	11.51 ± 1.24^a^
His	6.42 ± 0.42^a^	6.80 ± 0.67^a^	6.04 ± 0.52^a^	6.60 ± 0.58^a^	6.93 ± 0.73^a^	6.70 ± 0.63^a^
Ile	1.60 ± 0.08^a^	1.26 ± 0.11^b,c^	1.40 ± 0.19^a,b^	1.63 ± 0.11^a^	1.32 ± 0.10^b,c^	1.37 ± 0.09^b^
Lys^*^	**12.33 ± 1.20**^b^	9.65 ± 0.94^c^	8.91 ± 0.84^c,d^	9.17 ± 0.95^c,d^	10.06 ± 1.17^c^	**14.60 ± 1.22**^a^
Leu	5.90 ± 0.64^a^	6.25 ± 0.40^a^	6.20 ± 0.55^a^	5.87 ± 0.55^a^	5.82 ± 0.60^a^	5.68 ± 0.51^a^
Met	0.50 ± 0.04^a^	0.44 ± 0.08^a^	0.45 ± 0.06^a^	0.43 ± 0.08^a^	0.48 ± 0.07^a^	0.49 ± 0.11^a^
Pro^*^	**4.94 ± 0.45**^a^	2.69 ± 0.24^c^	2.54 ± 0.35^c^	2.00 ± 0.14^d^	2.95 ± 0.17^b,c^	3.33 ± 0.28^b^
Arg^*^	**6.83 ± 0.45**^a^	3.70 ± 0.15^c^	3.80 ± 0.18^c^	3.77 ± 0.21^c^	3.67 ± 0.32^c^	4.81 ± 0.45^b^
Ser^*^	**2.20 ± 0.12**^a^	1.21 ± 0.16^c^	1.47 ± 0.30^b,c^	1.57 ± 0.10^b^	1.43 ± 0.10^c^	1.40 ± 0.09^c^
Thr^*^	**1.10 ± 0.07**^a^	0.78 ± 0.08^c^	0.64 ± 0.04^c,d^	0.59 ± 0.05^d^	0.76 ± 0.07^c^	1.02 ± 0.04^a,b^
Val	2.92 ± 0.25^a^	2.37 ± 0.30^a,b^	2.43 ± 0.26^a,b^	2.65 ± 0.38^a,b^	2.50 ± 0.22^a,b^	3.23 ± 0.27^a^
Tyr	6.53 ± 0.48^a^	6.66 ± 0.59^a^	5.13 ± 0.48^b^	6.91 ± 0.62^a^	6.28 ± 0.56^a^	6.04 ± 0.40^a^

*shows significantly difference six amino acids content of Oryza sativa compared to five different gramineous species. Values labeled with different lowercase letters indicate a significant difference at *p* < 0.05 by Fisher’s protection least significant difference (PLSD)-test.

### Different Dietary Sources Influence Expression of TOR Pathway Genes

**Figure 1:**
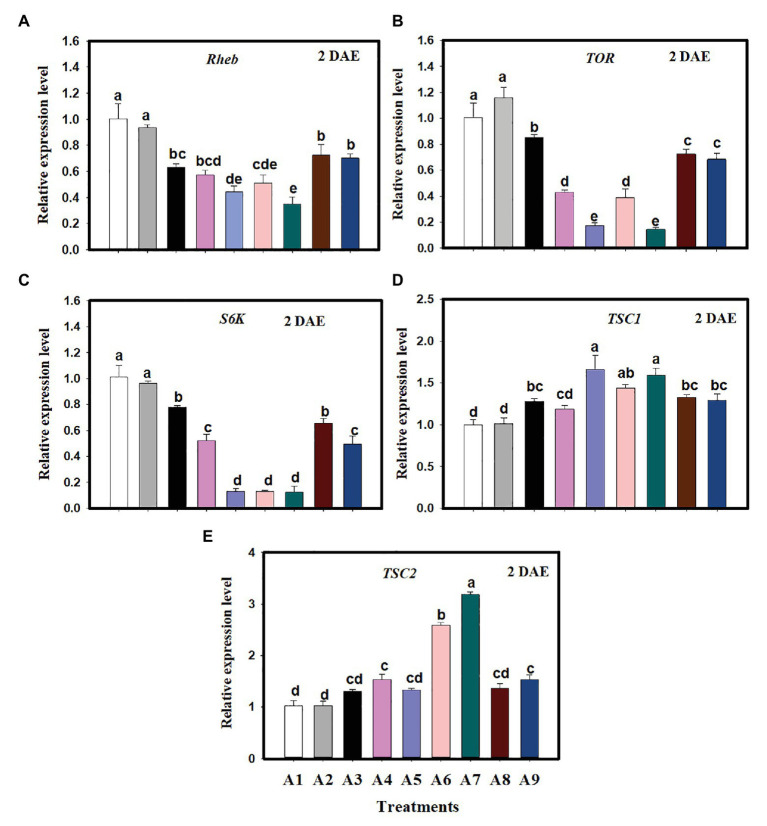
Expression of genes in the target of rapamycin (TOR) pathway in *Cyrtorhinus lividipennis* females supplied with various diets. Columns represent gene expression in *C. lividipennis* supplied with the following food sources: A1, BPH eggs on *Oryza sativa* (rice plant); A2, *Corcyra cephalonica* eggs; A3, *O. sativa* alone; A4, no food source (starvation); A5, *Cynodon dactylon* with BPH eggs; A6, *Digitaria ciliaris* with BPH eggs; A7, *Eleusine indica* with BPH eggs; A8, *Echinochloa glabrescens* with BPH eggs; and A9, *Leptochloa chinensis* with BPH eggs. Panels **(A–E)** show expression of *Rheb*, *TOR*, *S6K*, *TSC1*, and *TSC2* as measured by qPCR 2 days after emergence (DAE); β-actin was used as a reference gene for normalizing the data. Each treatment and control contained three replicates, and error bars represent the mean ± SEM. Columns labeled with different letters indicate a significant difference at *p* < 0.05 by the Student’s *t*-test.

Expression of selected genes in the TOR pathway was evaluated in newly emerged *C. lividipennis* females supplied with various food sources. When compared to *C. lividipennis* feeding on BPH eggs from rice, the expression of several TOR pathway genes was decreased when diets consisted of gramineous plants with BPH eggs, rice plants alone, or no food (starvation). For example, *Rheb* expression was decreased by 37–65% (Figure 1A; *F* = 38.4, *df* = 8, 26, *p* = 0.0001), down by 15–86% for *TOR* (Figure 1B; *F* = 205.9, *df* = 8, 26, *p* = 0.0001), and down by 23–88% for *S6K* (Figure 1C; *F* = 169.7, *df* = 8,26, *p* = 0.0001). In contrast, gene expression was 28–68% higher for *TSC1* (Figure 1D; *F* = 25.5, *df* = 8,26, *p* = 0.0001) and 30–210% higher for *TSC2* (Figure 1E; *F* = 82.5, *df* = 8,26, *p* = 0.0001) compared to *C. lividipennis* feeding on the BPH eggs from rice plants and *C. cephalonica* eggs. In general, there was no significant difference in the expression of TOR pathway genes in *C. lividipennis* females feeding on *C. cephalonica* eggs and BPH eggs inhabiting rice at 2 DAE (columns A1, A2; Figures 1A–E).

### TOR Gene Expression in Response to Amino Acid Signals

**Figure 2:**
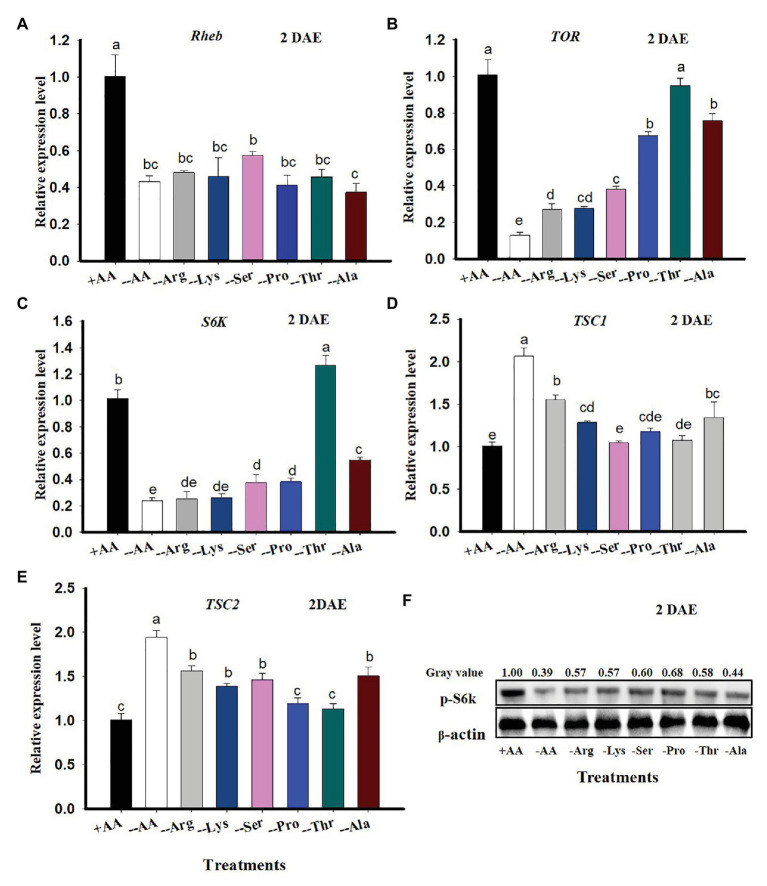
TOR gene expression in *C. lividipennis* in response to amino acid (AA) signals. Columns represent gene expression in *C. lividipennis* supplied with an artificial diet containing the following AAs:+AAs (+Ala, +Arg, +Lys, +Ser, +Pro, and +Thr), without −AAs (−6 AAs: −Ala, −Arg, −Lys, −Ser, −Pro, and −Thr), or lacking individual AAs (−1 AA: −Ala, −Arg, −Lys, −Ser, −Pro, or −Thr). Panels **(A–E)** show mean expression levels of *Rheb*, *TOR*, *S6K*, *TSC1*, and *TSC2* as measured by qPCR at DAE; β-actin was used as a reference gene. Panel **(F)** shows the phosphorylation status of S6K in adult females (*n* = 15) by western blot analysis. Antiserum to β-actin was used as a loading control. Each treatment and control represent three independent biological replicates, and error bars show means ± SEM. Columns labeled with different letters indicate a significant difference at *p* < 0.05 by the Student’s *t*-test.

When *C. lividipennis* females were supplied with artificial diets lacking six AAs, expression of selected TOR genes was significantly reduced. For example, *Rheb* expression was reduced by 57% (*F* = 11.4, *df* = 7,23, *p* = 0.0001), *TOR* was down by 87% (*F* = 64.7, *df* = 7, 23, *p* = 0.0001), and *S6K* was reduced by 76% (*F* = 77.0, *df* = 7, 23, *p* = 0.001) relative to females supplied with all AAs (Figures 2A–C). In contrast, expression of *TSC1* was 104% higher with the −AA diet (*F* = 56.5, *df* = 7, 23, *p* = 0.0001; Figure 2D) and *TSC2* was up by 93% (*F* = 53.6, *df* = 7,23, *p* = 0.0001; Figure 2E) as compared to females feeding on the +AA diet at 2 DAE.

When *C. lividipennis* females were deprived of individual AAs, expression of *Rheb*, *TOR*, and *S6K* was reduced by 52, 73, and 75% in Arg-deprived; 54, 73, and 74% in Lys-deprived; 43, 62, and 63% in Ser-deprived; 59, 33, and 62% in Pro-deprived, 54% in Thr- deprived (*Rheb* only); and 63, 25, and 46% in Ala-deprived groups as compared to those feeding on the complete diet (+AAs) at 2 DAE, respectively (Figures 2A–C). There was no significant difference in *TOR* expression levels in *C. lividipennis* females feeding on a Thr-deprived artificial diet and those feeding on a diet supplemented with all AAs (Figure 2B). *S6K* expression was upregulated by 25% in females fed on Thr-deprived artificial diets relative to those fed on the complete +AA artificial diet (Figure 2C).

*Cyrtorhinus lividipennis* females fed on diets lacking certain AAs led to upregulated *TSC1* and *TSC2*; expression was up by 54 and 55% in Arg-deprived, 27 and 38% in Lys-deprived, 45 and 0% in Ser-deprived (*TSC2*), and 33 and 32% in Ala-deprived groups as compared to females reared on all AAs, respectively (Figures 2D,E). There were no significant differences in *TSC1* or *TSC2* expression levels in females feeding on Pro- or Thr-deprived artificial diets (Figures 2D,E). *TSC1* expression was not significantly different in the Ser-deprived diet relative to the complete diet (Figure 2D). There was a noticeable decrease in S6K phosphorylation when *C. lividipennis* females were deprived of one or more AAs (Figure 2E).

### TOR Pathway Transduces AA Signals to Regulate Vg Synthesis

**Figure 3:**
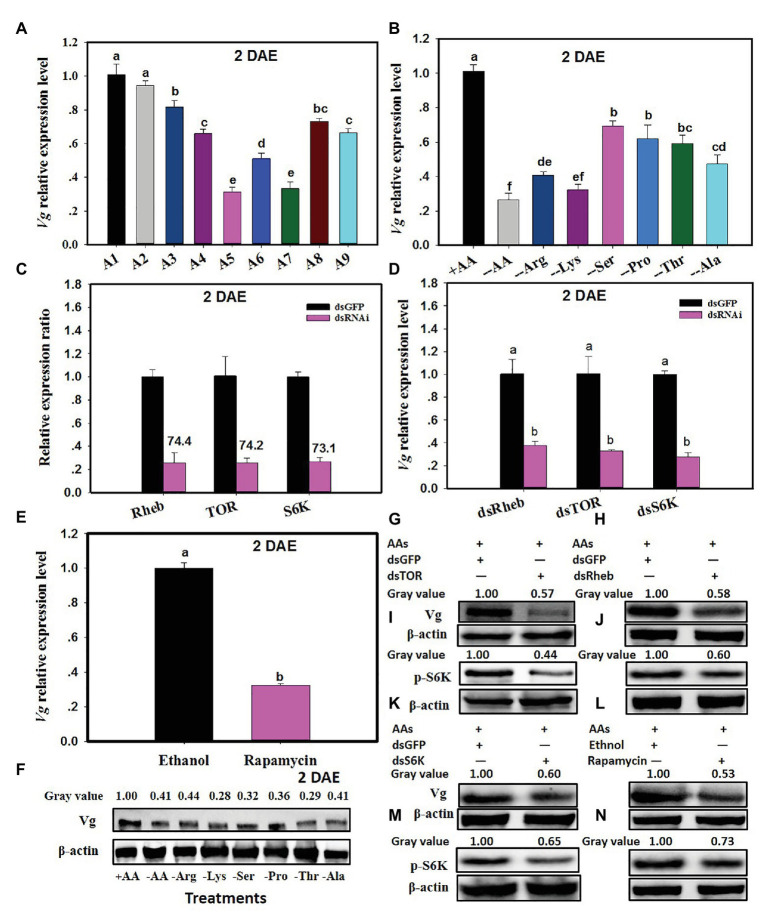
The TOR pathway transduces AA signals and regulates vitellogenin (Vg) synthesis. Panel **(A)** shows *Vg* expression in *C. lividipennis* females supplied with diets A1–A9 (as defined in Figure 1 legend); **(B)** shows *Vg* expression on artificial diets that vary in AAs (as defined in Figure 2 legend). Panel **(C)** illustrates the relative expression ratios of *Rheb*, *TOR*, and *S6K* in *C. lividipennis* females treated with dsRheb, dsTOR, and dsS6K, respectively. dsGFP was transfected as a negative control. Panel **(D)** shows mean *Vg* expression in *C. lividipennis* females treated with dietary dsRheb, dsTOR, and dsS6K. Panel **(E)** shows Vg expression in *C. lividipennis* females treated with rapamycin and ethanol (control). Panel **(F)** shows the Vg protein levels in adult females (*n* = 15) as determined by western blots with anti-Vg antiserum. Each treatment and control represent three independent biological replicates, and error bars show means ± SEM. Different lowercase letters in the histogram indicate significant differences at *p* < 0.05 (Student’s *t*-test). Panels **(G–N)** show Vg protein levels and pS6K phosphorylation status in *C. lividipennis* treated with dsRheb, dsTOR, dsS6K, and rapamycin. Proteins were detected with anti-Vg or anti-pS6K antisera and visualized by western blot analysis. Antiserum to β-actin was used as a loading control.

*Vitellogenin* expression in *C. lividipennis* was 27.3–69.1% lower when diets consisted of gramineous plants with BPH eggs, rice plants alone, or no food (starved) as compared to females feeding on BPH eggs from rice plants or *C. cephalonica* eggs (Figure 3A; *F* = 144.6, *df* = 8, 26, *p* = 0.0001). When *C. lividipennis* females were fed on diets lacking one or more AAs, *Vg* expression was down by 31–74% as compared to females fed on a complete artificial diet at 2 DAE (Figure 3B; *F* = 220.9, *df* = 7, 23, *p* = 0.0001). Western blot analysis confirmed that feeding on multiple or single AA-deprived artificial diets resulted in a significant reduction of Vg protein (Figure 3F).

The efficiency of silencing *Rheb*, *TOR*, and *S6K* was approximately 74.4, 74.2, and 73.1% at 2 DAE, respectively (Figure 3C). *Cyrtorhinus lividipennis* females treated with dsRheb, dsTOR, and dsS6K showed a 70% reduction in Vg expression as compared to the dsGFP control (Figure 3D). Western blots confirmed that S6K phosphorylation and Vg protein levels were reduced in dsRheb-, dsTOR-, and dsS6K-treated females (Figures 3G–M).

Rapamycin treatment also reduced *Vg* expression levels, which were 68% lower than females treated with the ethanol control (Figure 3E; *F* = 390.3, *df* = 1,5, *p* = 0.0001). Western blot analysis showed that both Vg levels and S6K phosphorylation were reduced in the rapamycin-treated females relative to the ethanol control (Figure 3N).

### TOR Pathway Functions *via* JH to Regulate Vg Synthesis

**Figure 4:**
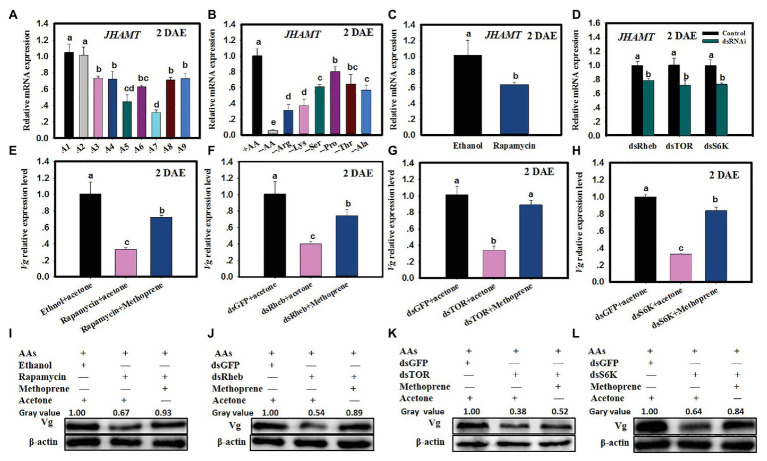
The TOR pathway functions *via* JH to regulate Vg synthesis. **(A)** *JHAMT* expression in *C. lividipennis* females supplied with diets A1–A9 (as defined in Figure 1 legend). **(B)** *JHAMT* expression in females supplied with artificial diets that vary in AAs (as defined in Figure 2 legend). **(C)** *JHAMT* expression in females treated with rapamycin and ethanol (control). **(D)** *JHAMT* expression in *C. lividipennis* treated with dietary dsRheb, dsTOR, and dsS6K. **(E)** *Vg* expression in females treated with 0.2 nM rapamycin and rapamycin with methoprene (100 ng/nl); the ethanol + acetone treatment served as solvent control. Panels **(F–H)** show Vg expression in females fed on dietary dsRheb, dsTOR, and dsS6K and exposed to methoprene or acetone. Females treated with dietary dsGFP and acetone served as a control. Panels **(I–L)** show Vg protein levels in *C. lividipennis* (*n* = 15) treated with rapamycin, methoprene, dsRheb, dsTOR, and dsS6K. Proteins were detected with anti-Vg antibodies and visualized by western blot analysis; antiserum to β-actin was used as a loading control. Each treatment and control consisted of three independent biological replicates. Error bars represent means ± SEM. Columns labeled with different lowercase letters indicate significant differences at *p* < 0.05 by Student’s *t*-test.

When *C. lividipennis* were fed on gramineous plants with BPH eggs, rice plants without BPH eggs, or no food (starvation), *JHAMT* expression was down by 28–70% as compared to females feeding on BPH eggs from rice plants or *C. cephalonica* eggs (Figure 4A; F = 35.4, *df* = 8,26, *p* = 0.0001). When *C. lividipennis* females were deprived of multiple or individual AAs, *JHAMT* expression levels were down by 20–94% as compared to females feeding on a complete artificial diet (Figure 4B; *F* = 91.4, *df* = 7,23, *p* = 0.0001). Rapamycin treatment significantly decreased *JHAMT* expression (down 37%) as compared to ethanol-treated control females (Figure 4C; *F* = 10.9, *df* = 1, 5, *p* = 0.0298). In dsRheb-, dsTOR-, and dsS6K-treated females, *JHAMT* expression levels were down by 22, 34, and 32% as compared to dsGFP treatments (Figure 4D; *F* = 34.3, *df* = 1, 5, *p* = 0.0043; *F* = 24.6, *df* = 1,5, *p* = 0.0077; *F* = 62.2, *df* = 1, 5, *p* = 0.0001).

The application of methoprene to *C. lividipennis* females treated with rapamycin, dsTOR, dsRheb, or dsS6K was partially rescued by *Vg* expression. For example, *Vg* expression was 117% higher when methoprene was added to rapamycin-treated females as compared to rapamycin alone (Figure 4E; *F* = 48.0, *df* = 2,8, *p* = 0.0002), upregulation was 86% higher when methoprene was added to dsRheb-treated females (Figure 4F; *F* = 143.6, *df* = 2,8, *p* = 0.0001), 156% higher as compared to dsTOR-treated females (Figure 4G; *F* = 102.6, *df* = 2,8, *p* = 0.0001), and 132% higher as compared to dsS6K-treated females (Figure 4H). Western blots confirmed the partial or full restoration of Vg production by methoprene in rapamycin, dsRheb-, dsTOR-, or dsS6K-treated females (Figures 4I–L).

**Figure 5:**
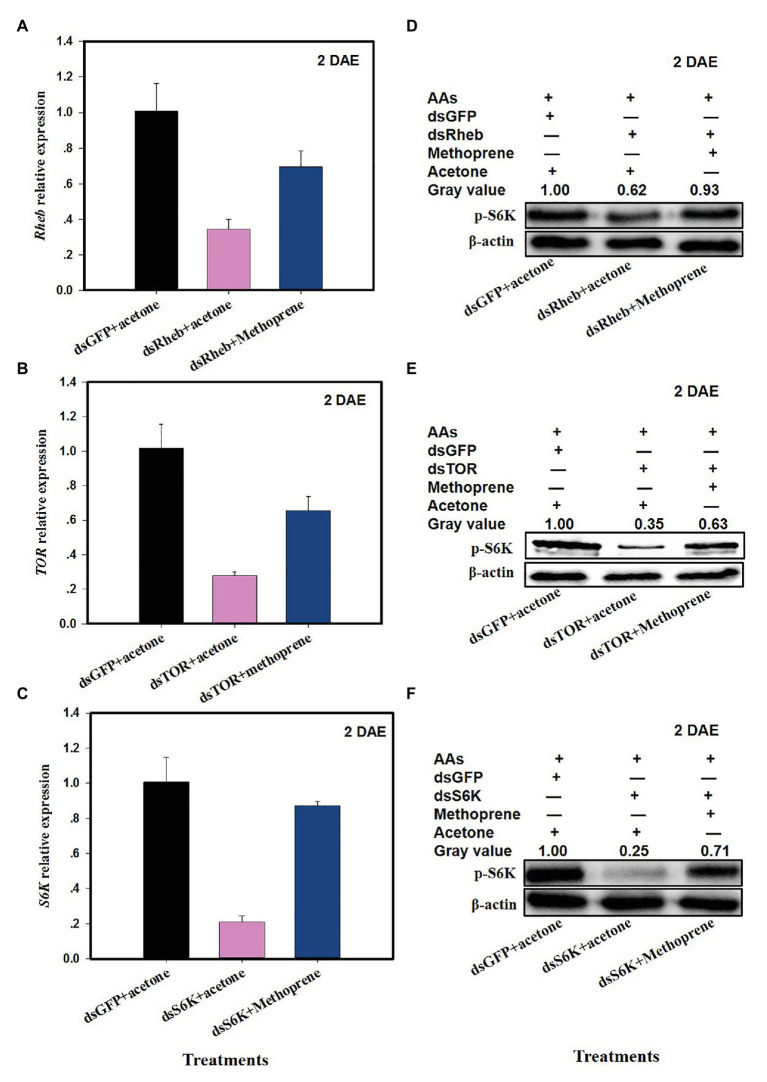
Effects of JH analog (JHA) methoprene on the expression of TOR pathway genes after dsRNA treatment at 2 DAE. Panels **(A)** *Rheb* expression in dsRheb- and methoprene-treated *C. lividipennis* females; **(B)** *TOR* expression in dsTOR and methoprene-treated *C. lividipennis* females; and **(C)** *S6K* expression in dsS6K and methoprene treated females; dsGFP and acetone were included as negative controls. Detection of phosphorylated SK6 in *C. lividipennis* treated with **(D)** methoprene and dsRheb; **(E)** methoprene and dsTOR, and **(F)** methoprene and dsSK6. Proteins were detected with anti-pS6K antiserum and visualized by western blot analysis. Antiserum to β-actin was used as a loading control. Each treatment and control consisted of three independent biological replicates. Error bars represent means ± SEM.

The addition of methoprene to dsRheb-, dsTOR-, or dsS6k-treated females partially restored *Rheb* (Figure 5A; *F* = 28.1, *df* = 2,8, *p* = 0.0009, up 103% as compared to dsRheb+acetone), *TOR* (Figure 5B; *F* = 16.0, *df* = 2,8, *p* = 0.0039, up 135% vs. dsTOR = acetone), or *S6K* (Figure 5C; *F* = 75.5, *df* = 2,8, *p* = 0.0001, up 315% vs. dsS6K + acetone). Western blots confirmed the partial or full restoration of S6K protein levels by exogenous methoprene in dsRheb-, dsTOR-, or dsS6K-treated females (Figures 5D–F).

### TOR Pathway Regulates Physiological and Reproductive Parameters in *C. lividipennis*

Treatment of *C. lividipennis* with dietary dsTOR resulted in multiple physiological changes relative to dsGFP at 2 DAE. These included a reduction in soluble protein content in ovaries (down 48%, Supplementary Figure S1A; *F* = 373.4, *df* = 1, 5, *p* = 0.0001) and fat bodies (down 38%, Supplementary Figure S1B; *F* = 19.8, *df* = 1, 5, *p* = 0.0112); furthermore, JH titers were reduced (down 33%, Supplementary Figure S1C; *F* = 106.2, *df* = 1, 5, *p* = 0.0003). Dietary dsTOR treatment also led to reduced female longevity (down 20%, Supplementary Figure S1E), body weight (down 31%, Supplementary Figure S1F), and predatory capacity at 1 DAE (down 44%, Supplementary Figure S1G, *F* = 10.3, *df* = 1, 19, *p* = 0.0001) and 3 DAE (down 27%, Supplementary Figure S1H, *F* = 6.3, *df* = 1, 19, *p* = 0.0001). Interestingly, dietary dsTOR treatment led to increased ecdysteroid titers (up 43%, Supplementary Figure S1D, *F* = 98.3, *df* = 1, 5, *p* = 0.0006).

**Figure 6:**
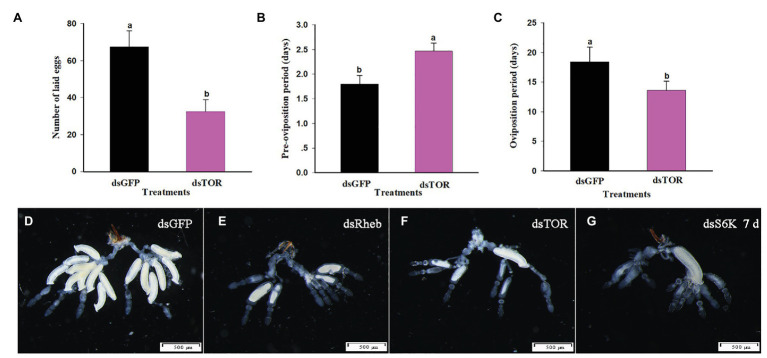
TOR pathway regulates reproductive parameters in *C. lividipennis* females. **(A)** Number of eggs laid, **(B)** preoviposition period, and **(C)** oviposition period for females treated with dsGFP and dsTOR. Histogram shows means ± SEM, and columns labeled with different lowercase letters were significantly difference at *p* < 0.05 using the Student’s *t*-test. Each treatment and control were from 15 independent biological replicates. Structural changes were shown in the ovaries of *C. lividipennis* females subjected to silencing of TOR pathway-related genes at 7 DAE. Representative images from dsGFP-treated control females **(D)**; and dsRheb- **(E)**, dsTOR- **(F)**, and dsS6K- **(G)** treated females. Reproductive tracts were dissected from at least 10 females from each group and photographed with a Leica DMR connected to a Fuji FinePix S2 Pro digital camera (Tokyo, Japan). Scale bar, 500 μm.

**Figure 7:**
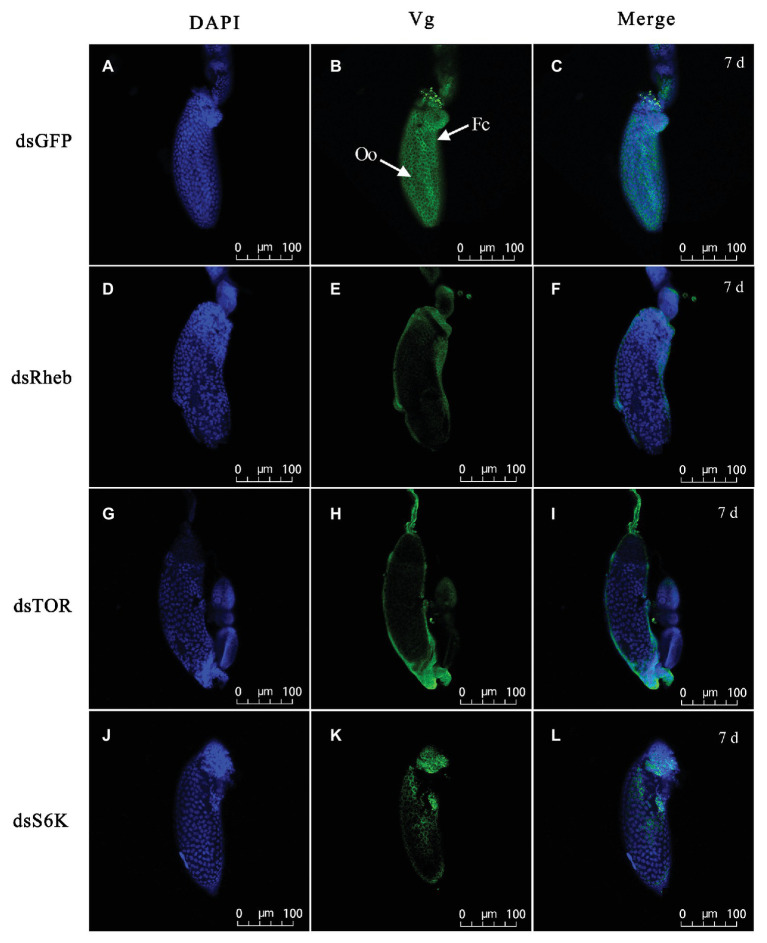
Silencing of TOR pathway-related gene changes Vg accumulation in ovarioles of adult females at 7 DAE. Left panels **(A,D,G,J)** show DAPI-stained nuclei; middle panels **(B,E,H,K)** show Vg protein detected with goat anti-rabbit IgG-labeled with Dylight 488 (green); right panels **(C,F,I,L)** showed merged images. Fluorescent images were captured with a Zeiss LSM 780 confocal microscope (Carl Zeiss MicroImaging, Göttingen, Germany). Fc, follicular cell; Oo, oocyte. Bars, 100 μm.

Treatment with dietary dsTOR caused reproductive changes in *C. lividipennis* relative to dsGFP. These included a reduction in the number of eggs laid (down 52%, Figure 6A, *F* = 23.1, *df* = 1, 29, *p* = 0.0001), a prolonged preoviposition period (up 37%, Figure 6B, *F* = 7.7, *df* = 1,29, *p* = 0.0098), and a reduced oviposition period (down 26%, Figure 6C, *F* = 39.9, *df* = 1, 29, *p* = 0.0001). Females treated with dsRheb, dsTOR, or dsS6K exhibited stunted, undeveloped ovaries (Figures 6E–G) and the absence of Vg staining in ovarioles of adult females compared to dsGFP treatment (Figure 7). These results were consistent with the changes in gene expression and protein production observed in dsRheb-, dsTOR-, or dsS6K-treated females.

### TOR Pathway Regulates Number of Offspring, Hatching Rate, and PGI

Dietary dsTOR resulted in reduced offspring number (*F1*), hatching rate, and PGI in females, which were down by 46, 14, and 37% as compared to the dsGFP treatment group, respectively (Table 2). Interestingly, dsTOR treatment had no impact on the gender ratio (Table 2).

**Table 2:** Effects of dietary dsTOR and dsGFP (control) on offspring number, hatching rate, gender ratio, and population growth index (PGI).

Treatments*^a^*	Number of offspring*^b^*	Hatching rate*^c^*	Sex ratio*^c^*	PGI (N1/N0)*^d^*
dsGFP	209.8 ± 13.6^a^	0.78 ± 0.04^a^	1.10 ± 0.06^a^	33.8 ± 2.72^a^
dsTOR	114.0 ± 12.4^b^	0.67 ± 0.05^b^	1.01 ± 0.06^a^	21.2 ± 1.89^b^

a Treatments included: dsTOR treated females mated with untreated males (treatment group); and dsGFP-treated females mated with untreated males (control group).

b Values labeled with different letters indicate a significant difference at *p* < 0.05 by the Student’s *t*-test.

c Hatch rates and sex ratios were calculated as described by Ge et al. (2019).

d The PGI was expressed by the ratio N1/N0; this was determined by dividing the total number (N1) of adults of next generation + unhatched eggs by the number of adults released (N0 = 8, 4 pairs).

## Discussion

Nutrients play a critical role in regulating insect reproduction (Attardo et al., 2005). Earlier studies demonstrated that a diet of gramineous plants containing eggs of BPH resulted in reduced development and reproduction in *C. lividipennis* compared to insects feeding on BPH eggs from rice plants or *C. cephalonica* eggs (Yu et al., 1996). However, the regulatory mechanism of this phenomenon was unclear. The present study shows that rice plants contain higher concentrations of Ala, Lys, Pro, Arg, Ser, and Thr than the five gramineous species (Table 1), suggesting that these six AAs might be associated with development and reproduction of *C. lividipennis*. We also found that *TOR* was significantly upregulated when *C. lividipennis* was supplied rice plants containing BPH eggs as compared to gramineous species with eggs based on transcriptomic data (data not shown). Using a defined artificial diet (Ge et al., 2020b), we infer that the TOR signaling pathway senses these six AA signals and regulates downstream genes and proteins, thus leading to an increase in Vg synthesis, reproduction, and population growth of *C. lividipennis*.

Target of rapamycin is a serine/threonine kinase that consolidates nutritional signals *via* AAs (Kim and Guan, 2011). The disruption of the TOR signaling pathway has been associated with the reduced reproductive capacity that occurred when nutrients were limiting in *Tribolium castaneum*, *B. germanica*, and *A. aegypti* (Hansen et al., 2004, 2005; Parhasarathy and Palli, 2011). When *C. lividipennis* was supplied with a diet of gramineous plants plus BPH eggs or rice plants alone, multiple genes in the TOR pathway were repressed (Figure 1), and similar results were obtained with diets lacking one or multiple AAs (Figure 2). The TOR signaling pathway regulates multiple cellular functions including proliferation, apoptosis, growth, and autophagy by integrating a multitude of nutritional signals (Yang et al., 2006). The tumor suppressors, TSC1 and TSC2, function in both *Drosophila* and mammals as negative regulators of *Rheb* (Fingar and Blenis, 2004; Pan et al., 2004; Yang et al., 2006). TORC1, which is sensitive to rapamycin, modulates cellular growth and protein biogenesis by phosphorylating the translational regulators 4EBP1 and S6K (Kwiatkowski, 2003). Previous research demonstrated that AA-dependent nutritional signaling mediates S6K phosphorylation in the TOR signaling pathway (Maestro et al., 2009). In this study, RNAi-mediated knockdown of *Rheb*, *TOR*, and *S6K* led to repressed expression of *Vg* and reduced Vg synthesis and a decline in S6K phosphorylation (Figure 3). Similarly, the TOR inhibitor rapamycin also led to reduced levels of Vg and S6K phosphorylation in *C. lividipennis* at 2 DAE (Figure 3).

Target of rapamycin is known to transduce nutritional signals in many organisms including vertebrates, invertebrates, and yeast (Wang and Pround, 2009; González and Hall, 2017). In the red flour beetle, *T. castaneum*, the two primary regulators of *Vg* transcription were JH and nutrition (Parthasarathy et al., 2010; Sheng et al., 2011). With respect to JH, we showed that *JHAMT* expression was reduced when *C. lividipennis* was fed on the gramineous plants containing *N. lugens* eggs or supplied with diets lacking one or more of six identified AAs. RNAi-mediated knockdown of the TOR pathway genes (*Rheb*, *TOR*, and *S6K*) or injection with the TOR inhibitor rapamycin also resulted in a dramatic reduction in *JHAMT* expression (Figure 4). Thus, the TOR signaling pathway in *C. lividipennis* regulated JH biosynthesis in response to AA signals. Furthermore, the application of the JHA methoprene to *C. lividipennis* females injected with rapamycin or treated with dsRheb, dsTOR, or dsS6K partially rescued *Vg*, *Rheb*, *TOR* and *S6K* expression, Vg levels, and S6K phosphorylation (Figures 4, 5). Therefore, we inferred that AA signals were transduced through the TOR pathway and regulated Vg synthesis through influencing JH biosynthesis in *C. lividipennis*. In female insects, JH participates in the regulation of vitellogenesis (Fronstin and Hatle, 2008; Shiao et al., 2008), and the regulation of JH *via* nutritional signals and the TOR pathway is well-established for several insect species (Tu et al., 2005; Sheng et al., 2011; Perex-Hedo et al., 2013).

Target of rapamycin has a critical function in the development of insect reproductive systems (Park et al., 2006). The synthesis of Vg and its uptake by oocytes play a critical function in the invertebrate reproductive process (Tufail and Takeda, 2009). Results in our study demonstrate that dietary dsTOR causes a dramatic reduction in soluble proteins in ovaries and fat bodies, increases ecdysteroid titers, and decreases JH titers, female longevity, body weight, predatory capacity, number of laid eggs, and the oviposition period (Supplementary Figure S1 and Figure 6). Furthermore, RNAi-mediated knockdown of TOR pathway genes interfered with normal development of the female reproductive system and the uptake of Vg by oocytes (Figures 6, 7). dsTOR treatments also led to reduced numbers of offspring, declined hatching rates, and reduced PGI (Table 2), which is consistent with TOR function in tissue development, cell growth and proliferation, and nutritional signaling (Wullschleger et al., 2006). The process of vitellogenesis in many insects starts with the uptake of AAs and proteins (Hansen et al., 2004; Abrisqueta et al., 2014). The AA/TOR and insulin pathways function as nutritional sensors, impact reproductive organs, and modulate the biosynthesis of ecdysteroids and JH (Smykal and Raikhel, 2015). Ecdysteroids regulate vitellogenesis and the maturation of egg when nutritional resources are adequate (Gaziova et al., 2004; Terashima and Bownes, 2004). Previous studies demonstrated that RNAi-mediated knockdown of TOR expression in insects can decrease number of eggs laid and can impact ovary development and fecundity (Hansen et al., 2005; Lu et al., 2016; Liu et al., 2017; Zhuo et al., 2017).

In summary, this study shows that elevated concentrations of six AAs in rice plants function as key nutritional signals in JH and ecdysteroids biosynthesis, Vg biogenesis, ovary development, reproduction, and population growth in *C. lividipennis* (Supplementary Figure S2). The present study provides information on the AAs that foster reproduction of *C. lividipennis* illustrates the relationship between host nutrition and insect reproduction in predatory mirid bugs. It is important to note that plants produce many other secondary metabolites that could either promote or interfere with metabolism, neural transmission, or reproduction of herbivores (Wink, 1988, 2003). Investigations are underway to explore whether secondary metabolites present in gramineous species impact the reproduction and development of *C. lividipennis*, which are necessary steps in formulating an artificial diet that promotes longevity and fecundity of this important predator.

## Data Availability Statement

The original contributions presented in the study are included in the article/Supplementary Material, further inquiries can be directed to the corresponding authors.

## Author Contributions

LG wrote original draft and designed the experiment. HZ, SZ, and JX perform this experiment. QW performed the data analysis. QS modified the final manuscript. All authors contributed to the article and approved the submitted version.

### Conflict of Interest

The authors declare that the research was conducted in the absence of any commercial or financial relationships that could be construed as a potential conflict of interest.
