# Supplementary material for: The Amino Acid-Mediated TOR Pathway Regulates Reproductive Potential and Population Growth in Cyrtorhinus lividipennis Reuter (Hemiptera: Miridae)
Source: Front Physiol. 2020 Nov 30;11:617237. doi: 10.3389/fphys.2020.617237 (PMC7733968; doi:10.3389/fphys.2020.617237)
Supplement: Supplementary file 1 [file Data_Sheet_1.docx]

**
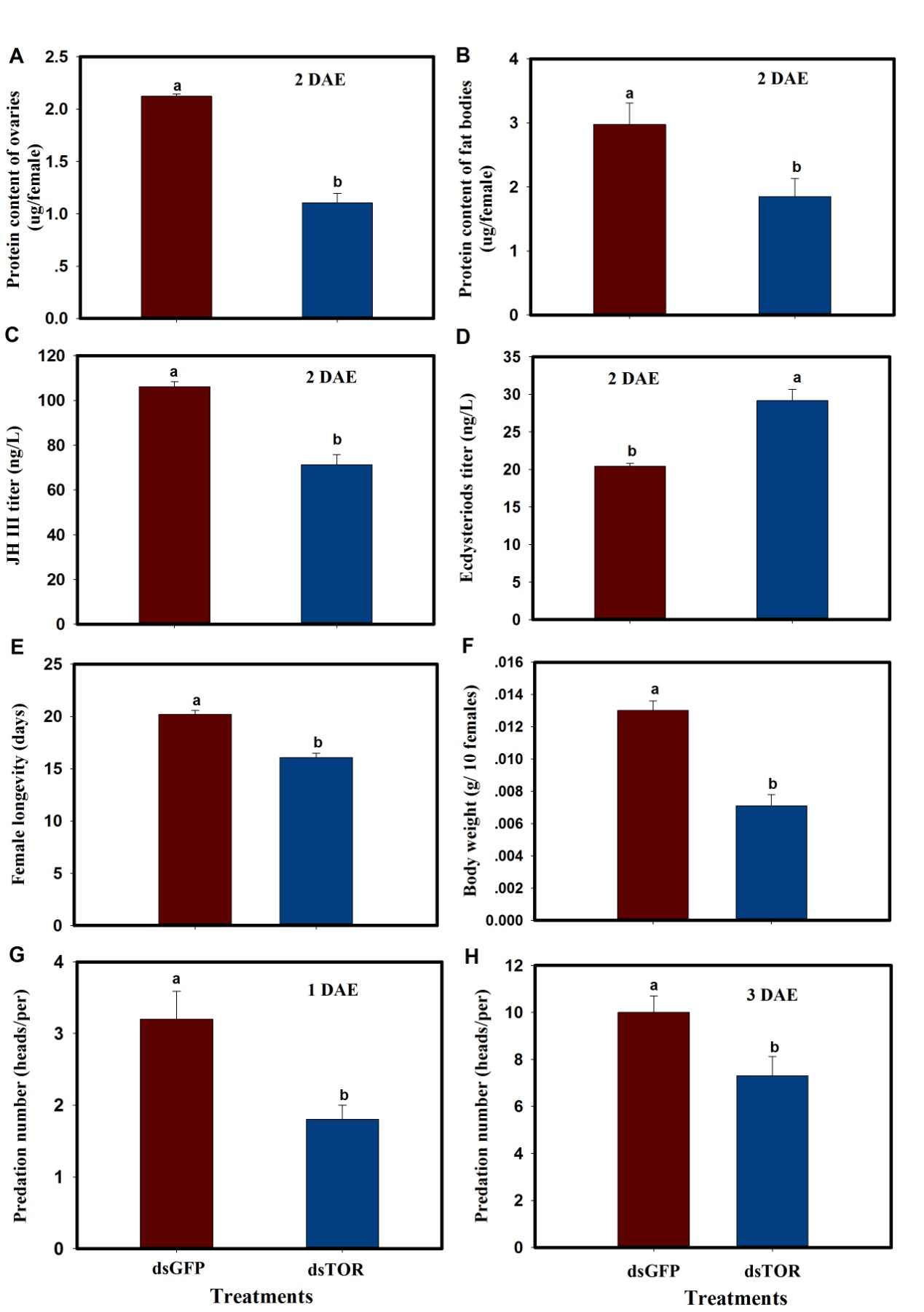
**

**Fig S1**  TOR pathway regulates physiological parameters in *C. lividipennis* females. Effect of dietary dsTOR treatment on (A) soluble protein content of ovaries, (B) soluble protein content of fat bodies, (C) JH titer, (D) ecdysteroid titer, (E) longevity of females (days), and (F) body weight (g/10 females) at 2 DAE. Panels (G) and (H) show predatory capacity at 1 DAE and 3 DAE. Error bars represent means ± SEM. Columns labeled with different lowercase letters indicate significant differences at *P*<0.05 by Student’s *t*-test. Each treatment and control consisted of three independent biological replicates or five independent biological replicates for predatory capacity.
