# Supplementary material for: The Amino Acid-Mediated TOR Pathway Regulates Reproductive Potential and Population Growth in Cyrtorhinus lividipennis Reuter (Hemiptera: Miridae)
Source: Front Physiol. 2020 Nov 30;11:617237. doi: 10.3389/fphys.2020.617237 (PMC7733968; doi:10.3389/fphys.2020.617237)
Supplement: Supplementary file 2 [file Data_Sheet_2.docx]

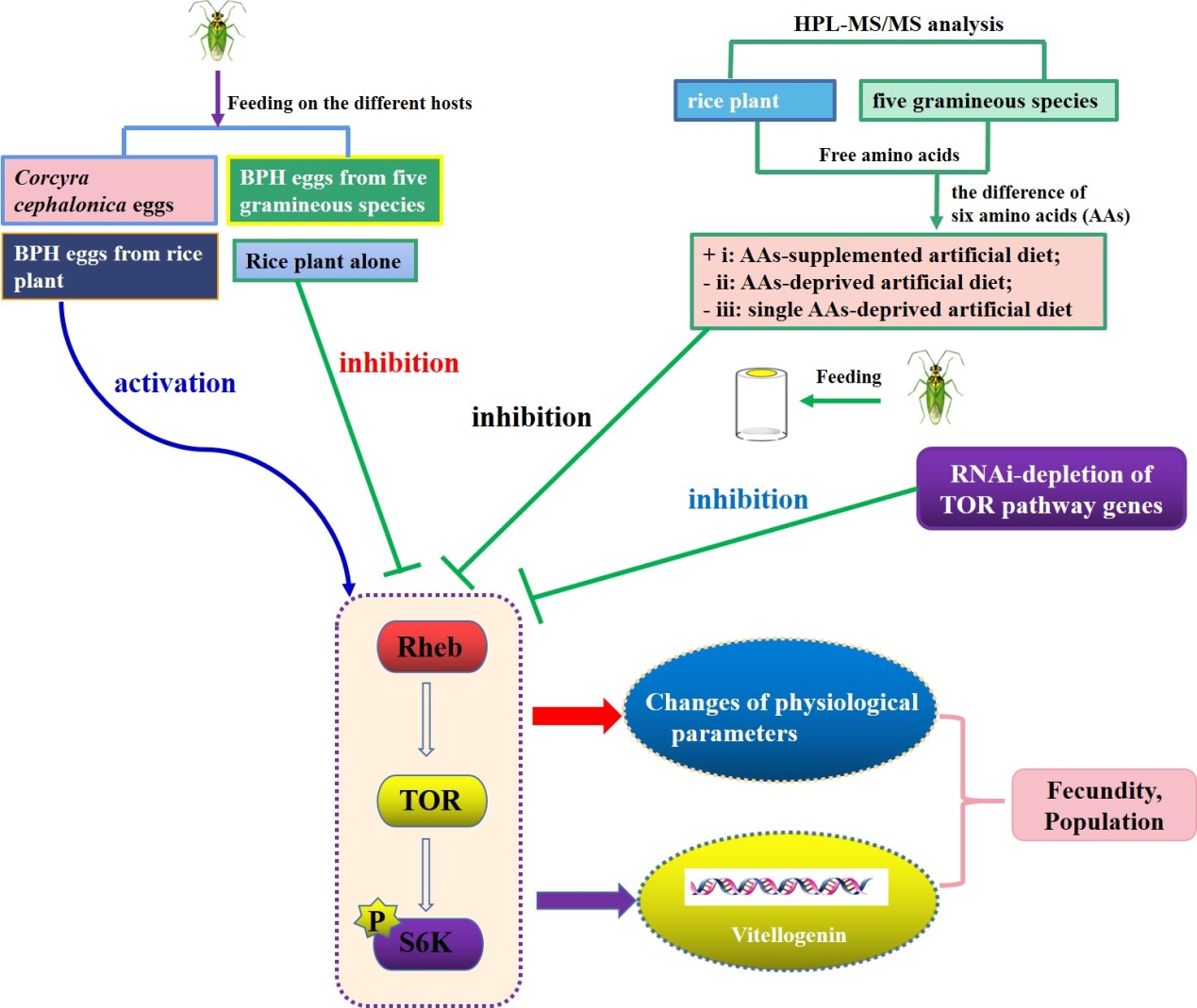


Fig S2 A proposed mode for TOR pathway response to six aminao acids in regulating *C. lividipennis* fecundity and population. *C. lividipennis* feeding BPH eggs from rice plant or *C. cephalonica* eggs activated the TOR pathway signal transuduction, while those feeding BPH eggs from five gramineous species or rice plant alone inhibited the TOR pathway signal transduction. Six free amino acid concentrations in rice plant was significantly higher than that of five graminous species by HPLC-MS/MS analysis. *C. lividipennis* feeding six AAs-deprived artificial diet or signle AA-deprive artificial diet or RNAi-depletion of *TOR* pathway genes lead to inhibition of the TOR signal pathway, resulting in significantly reduced reproduction and population growth.
