# Supplementary material for: The Amino Acid-Mediated TOR Pathway Regulates Reproductive Potential and Population Growth in Cyrtorhinus lividipennis Reuter (Hemiptera: Miridae)
Source: Front Physiol. 2020 Nov 30;11:617237. doi: 10.3389/fphys.2020.617237 (PMC7733968; doi:10.3389/fphys.2020.617237)
Supplement: Supplementary file 3 [file Table_1.docx]

Table S1. PCR primers used in this study.

| **GenBank No.** | **Primer ID** | **Sequences (5'-3')** | **Size (bp)** | **Tm°C** |
| --- | --- | --- | --- | --- |
|  | **qPCR analysis** |  |  |  |
| **MT360028** | QRheb-F | GGCAGTTCGTTGATTCCT | 101 | 57.6 |
|  | QRheb-R | CGCAGTGTCCACCAGTTT |  |  |
| **MT364788** | QTOR-F | ATCCCTCGAATTTGATGCTG | 203 | 58.1 |
|  | QTOR-R | AAGCACCTTCATCACGCTCT |  |  |
| **MT364789** | QS6K-F | GATGACGTCTCGCAGTTTGA | 173 | 55.1 |
|  | QS6K-R | CTCGGTGACCGTGCTTTTAT |  |  |
| **MT367206** | QTSC1-F | AGGGAAACTAACTCAGGACA | 88 | 52.8 |
|  | QTSC1-R | GAAATGGACGCACGAAAA |  |  |
| **MT367207** | QTSC2-F | AGTTGTCCAAAGACGGTTCG | 193 | 59.3 |
|  | QTSC2-R | CAACAAGCGCATACCTGATG |  |  |
| **MG996442** | QJHAMT-F | CCGTCTAAAGCCAAGGAA | 120 | 56.6 |
|  | QJHAMT-R | AGCGTGGACAGTCAAAGTAG |  |  |
| **KJ652904** | QVg-F | CCCTAGTCAGTCCAGTGCC | 192 | 61.2 |
|  | QVg-R | TGGCGAGAGGAACTGTCA |  |  |
| **EU179846** | Qβ-actin-F | CCGTCCCCATCTATGAAGGTT | 200 |  |
|  | Qβ-actin-R | GCGGTCGTCGTGAAGGAGTAAC |  |  |
|  | **dsRNA synthesis** |  |  |  |
| **MT360028** | Rheb-F | GGGCAGTTCGTTGATTCCTA | 380 |  |
|  | Rheb-R | GACTCATTGTGCTTGGCAGA |  |  |
|  | Rheb-T_7_F | taatacgactcactataggg (T_7_ promoter) GGGCAGTTCGTTGATTCCTA |  |  |
|  | Rheb-T_7_R | taatacgactcactataggg (T_7_ promoter) GACTCATTGTGCTTGGCAGA |  |  |
| **MT364788** | TOR-F | CCGGGAAAAGAAGAAAATCC | 463 |  |
|  | TOR-R | AAGCACCTTCATCACGCTCT |  |  |
|  | TOR-T_7_F | taatacgactcactataggg (T_7_ promoter) CCGGGAAAAGAAGAAAATCC |  |  |
|  | TOR-T_7_R | taatacgactcactataggg (T_7_ promoter) AAGCACCTTCATCACGCTCT |  |  |
| **MT364789** | S6K-F | TTCTACCGCCTTACCTGACG | 373 |  |
|  | S6K-R | CTCGGTGACCGTGCTTTTAT |  |  |
|  | S6K-T_7_F | taatacgactcactataggg (T_7_ promoter) TTCTACCGCCTTACCTGACG |  |  |
|  | S6K-T_7_R | taatacgactcactataggg (T_7_ promoter) CTCGGTGACCGTGCTTTTAT |  |  |
| **ACY56286** | GFP-F | AAGGGCGAGGAGCTGTTCACCG | 688 |  |
|  | GFP-R | CAGCAGGACCATGTGATCGCGC |  |  |
|  | GFP-T_7_F | taatacgactcactataggg (T7 promoter) AAGGGCGAGGAGCTGTTCACCG |  |  |
|  | GFP-T_7_R | taatacgactcactataggg (T7 promoter) CAGCAGGACCATGTGATCGCGC |  |  |
